# The Age of Young Nurses Is a Predictor of Burnout Syndrome during the Care of Patients with COVID-19

**DOI:** 10.3390/nursrep13020063

**Published:** 2023-04-17

**Authors:** Jeel Moya-Salazar, Liliana A. Buitrón, Eliane A. Goicochea, Carmen R. Salazar, Belén Moya-Salazar, Hans Contreras-Pulache

**Affiliations:** 1 Department of Pathology, Hospital Nacional Docente Madre Niño San Bartolomé, Lima 51001, Peru; 2 Facultad de Ciencias de la Salud, Universida Privada del Norte, Lima 51001, Peru; 3 School of Nursing, Faculties of Health Science, Universidad Norbert Wiener, Lima 51001, Peru; 4 Nursing Deparment, Hospital Nacional Dos de Mayo, Lima 51001, Peru; 5 School of Medical Technologist, Faculties of Health Science, Universidad Tecnológica del Perú, Lima 51001, Peru; 6 Qualitative Unite, Nesh Hubbs, Lima 51001, Peru; 7 South American Center for Research in Education and Public Health, Universidad Norbert Wiener, Lima 51001, Peru

**Keywords:** COVID-19, burnout syndrome, nurses, young adult, professional exhaustion, Peru

## Abstract

Background: Burnout Syndrome (BS) is a work fatigue phenomenon that leads to physical exhaustion during care work, and there could be an increase in the proportion of nurses affected during the COVID-19 pandemic, especially in those caring for infected patients. We aimed to determine BS in nurses during the COVID-19 pandemic. Methods: An observational study was conducted on 100 nurses over the age of 18 and working in COVID-19 medical units in 2021. The 22-item Maslach Burnout Inventory questionnaire was used to estimate BS, and differences between age groups, gender, work time, and previous infection were estimated. Results: The majority of nurses (mean 30 ± 5.5 years) were women (78%), and the most frequent working time was from 1 to 10 years (58%). A total of 88% of the nurses had moderate BS, affecting more males, aged between 20 and 30 years, and without previous infection. The youngest age group, 20–30 years, presented the highest mean BS with 53.8 (SD 4.18) points (95% CI: 52.79 to 54.8), showing differences with older nurses (*p* < 0.05). Prediction analysis showed that only age was a significant predictor for the development of SB (*p* < 0.001). Conclusions: BS negatively impacts young nurses during the care of COVID-19 patients, so strategies should be promoted to ensure a better working environment. Improving the workspace can include self-care strategies, changes in the system and work organization, an improvement of interpersonal relationships, and risk prevention.

## 1. Introduction

Work fatigue is the feeling of extreme tiredness resulting from long hours of heavy and monotonous work. In the absence of sleep, it can be counterproductive to physical and mental health. Among the most detrimental signs, symptoms, and effects of fatigue in the work environment, we have lethargy, a lack of motivation, decreased productivity, the inability to remember details or instructions, and the inability to concentrate and solve day-to-day work problems [1]. Likewise, the health sector is one of the most demanding jobs since it often consists of dealing with emergencies where people’s lives are at risk, for which reason healthcare workers (HCWs) are exposed to situations of prolonged stress and emotional load that can lead them to present health problems such as anxiety, depression, and sleep disorders [2]. This phenomenon of work fatigue that leads to physical exhaustion in nursing work is known as burnout syndrome (BS) [3].

In Latin America, the frequency of this BS varies between 2.1% and 76%, with higher rates in areas such as intensive care units (ICU), emergency rooms, and surgery [4]. The prevalence of BS-related outcomes was also reported, with doctors and nurses being most affected (44.4% and 32.3% of minor and moderate personal deterioration, respectively), and 49.3% experiencing little family deterioration [5]. In a systematic review conducted during the COVID-19 pandemic, the overall pooled prevalence of BS among HCWs was reported to be 52%, with 51% of HCWs being emotionally exhausted, 52% being depersonalized, and 28% having a low sense of personal accomplishment [6]. Another review showed that nurses had a specific global prevalence of 34.1% for emotional exhaustion, 12.6% for depersonalization, and 15.2% for lack of personal fulfillment [7].

Although the frequency of BS during the COVID-19 pandemic has not been quantified in Peru, previous studies have estimated that the incidence of BS among nursing professionals ranges from 2.8% to 50%, affecting all healthcare sectors [8,9]. This is understandable as they work long shifts, which affect their sleep cycles while they have to perform their duties optimally. Consequently, nurses who worked more than 60 h per week reported greater physical fatigue than nurses who worked 40 h or less [10].

The pandemic has dealt a blow to the Peruvian health system, as the strategies implemented to control and prevent COVID-19 were insufficient [11]. The rapid increase in infections and deaths during the first and second waves, the delay in the detection of early symptoms such as hypoxia, the lack of oxygen supply [12], as well as the lack of epidemiological tracking and control, have generated an overload for HCWs, impacting their health [13,14].

From the above, it can be understood why nursing professionals experience constant stress in the development of their work, especially in light of the health conditions affecting the globe during the pandemic [15]. In addition, some factors could exacerbate the development of complications derived from work overload and BS; these could be linked to the age group of frontline nursing professionals, the time they work in quarantine areas or are specialized in COVID-19, fewer resources available in health centers, and less specialized training against COVID-19 [7]. The COVID-19 pandemic has meant an overload of work for nursing professionals and may lead them to have the intention of leaving their profession, which harms the health system and patient care [16]. SB could be the trigger for this situation, which until now has not been well described in Latin America [6,7], especially in Peru, a country that has been hit hard by the pandemic.

We aimed to determine the prevalence of BS in the nursing staff of the COVID-19 medicine units, defining the factors that could predict its occurrence. The hypothesis of this study is that (i) BS mainly affects young nurses, (ii) working time is a predictive factor, and (iii) during the second outbreak of COVID-19, the dimension of emotional exhaustion was significantly affected.

## 2. Materials and Methods

### 2.1. Study Design and Settings

We used an observational survey-based study of 250 nurses from the COVID-19 medicine units at Hospital Dos de Mayo. The hospital, which is the first Peruvian public hospital managed by the Peruvian Ministry of Health, has more than 420 beds [17] and has reported more than 2580 patients positive with COVID-19 [18]. This study was conducted during the second wave of COVID-19 in 2021.

### 2.2. Population, Inclusion Criteria, and Instruments

**Figure 1:**
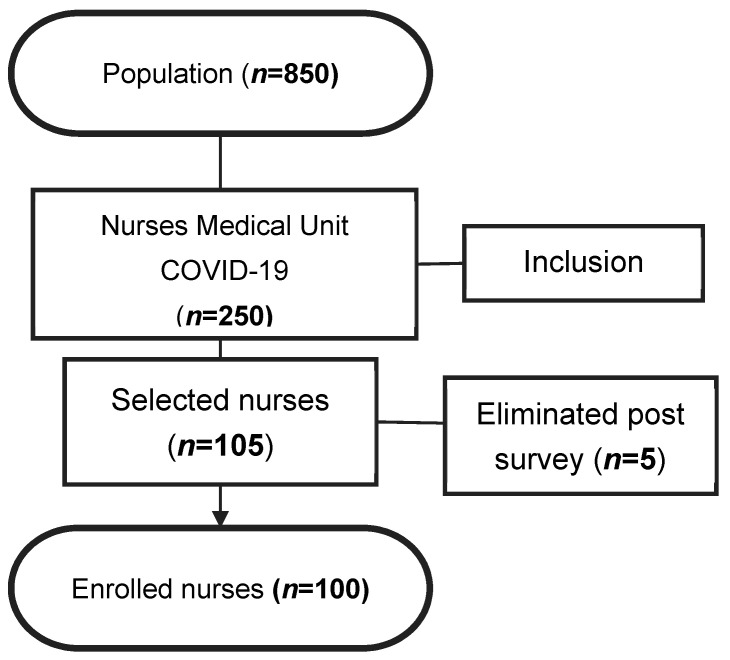
Nurse selection flowchart.

Inclusion criteria were male and female volunteer nurses older than 18 years working in the COVID-19 Medicine Unit. Nurses with chronic diseases, cancer, pregnant women, and tuberculosis were excluded. We also excluded paramedics with dual jobs and department heads. Of the total number of nurses at Hospital Dos de Mayo (*n* = 850), 250 worked in the care units for patients with COVID-19. We initially selected 105 nurses who completed the surveys, but we considered 100 nurses as a sample since they met the quality of survey completion (Figure 1).

We used the Maslach Burnout Inventory (MBI) questionnaire, which was created by Maslach and Jackson in 1981, to assess BS [19] in its Spanish version, validated by Gil-Monte and Peiró in 1997 [20]. The MBI has 22 items and covers three dimensions: exhaustion or emotional exhaustion (9 items), cynicism or depersonalization (5 items), and personal fulfillment or professional efficacy (8 items). MBI responses are presented on a 7-level frequency Likert-like scale such as never (0 points), a few times a year or less (1 point), once a month or less (2 points), a few times a month (3 points), once a week (4 points), a few times a week (5 points), and every day (6 points) [21]. According to the scores obtained, the frequency and intensity of the questionnaire measures are high from 67 to 132 points, medium from 34 to 66 points, and low from 1 to 33 points [22].

### 2.3. Variables, Data Processing and Analysis

The study variables were demographic characteristics (age, sex), work characteristics (work time), and clinical characteristics (previous COVID-19 infection). These surveys were conducted with prior coordination, and after completing the informed consent form, the average response time for each questionnaire was 10 min. Questionnaires were quality-checked and coded into a data matrix in MS Excel 2010 for Windows.

The data analysis had a descriptive approach with the estimation of simple and relative frequencies and the measures of central tendency (i.e., standard deviation—SD) according to the characteristics of the study variables. The MBI guidelines were followed to estimate the BS in each dimension, the scores, and the total work fatigue in nursing staff. In addition, after estimating the normality of the data with the Kolmogorov–Smirnov test, the non-paired Student’s *t*-test, and one-way ANOVA with Bonferroni post hoc test correction were used to demonstrate the difference between the groups of analysis by the length of service, hours of work, and history of infection. Finally, we performed a binary regression analysis to determine the predictive variables of BS, considering a *p*-value < 0.05 and a 95% confidence interval as significant for all the analyses.

### 2.4. Ethical Aspect

This study was approved by the ethics committees of the Dos de Mayo Hospital and the Universidad Norbert Wiener (Registration No. 152-2021). In addition, we followed the principles of bioethics and the guidelines of the Declaration of Helsinki [23].

## 3. Results

The average age of nursing staff is 30 (SD 5.5) years old (range: 20–50 years), with 20–30 years old being the most frequent (68%). The majority were women (78%), and the most frequent working time was from 1 to 10 years for 58%. A total of 88% of nurses exhibited moderate BS, affecting more males, aged 20 to 30, and not previously infected with SARS-CoV-2. On the other hand, severe symptoms of BS are seen in women over the age of 30 who have worked for 11 to 20 years.

A total of 88% of nurses presented moderate BS, affecting more men between 20 and 30 years of age, and without previous SARS-CoV-2 infection. On the other hand, severe symptoms of BS were found in women over the age of 30 who had worked for 11 to 20 years (Table 1). The three dimensions of SB showed, respectively, that 95% (95/100) of the participants reported low levels of emotional exhaustion, 57% (57/100) had low levels of depersonalization (but 34% (34/100) had moderate levels), and 76% (76/100) presented high levels of personal achievement. Furthermore, 59% (59/100) of the nurses were not infected with COVID-19.

In the item-by-item analysis of the first dimension (emotional exhaustion), 39% (39/100) of the nurses responded both “once a month or less” and “a few times a month” to “I feel emotionally exhausted by my work.” Additionally, 59% (59/100) responded “a few times a year or less” to “When I get up in the morning and face another day of work, I feel fatigued”.

For the depersonalization dimension, 63% (63/100) and 65% (65/100) responded as “never” to “I think I am treating some students as if they were impersonal objects” and “I don’t care really what happens to some of my students,” respectively. For the personal achievement dimension, 71% (71/100) responded “every day” to “I think I treat my students’ problems very effectively” and 58% (58/100) to “I think I get a lot of valuable things out of this job”. In total, 88% (88/100) of the nurses had a medium level of SB.

**Figure 2:**
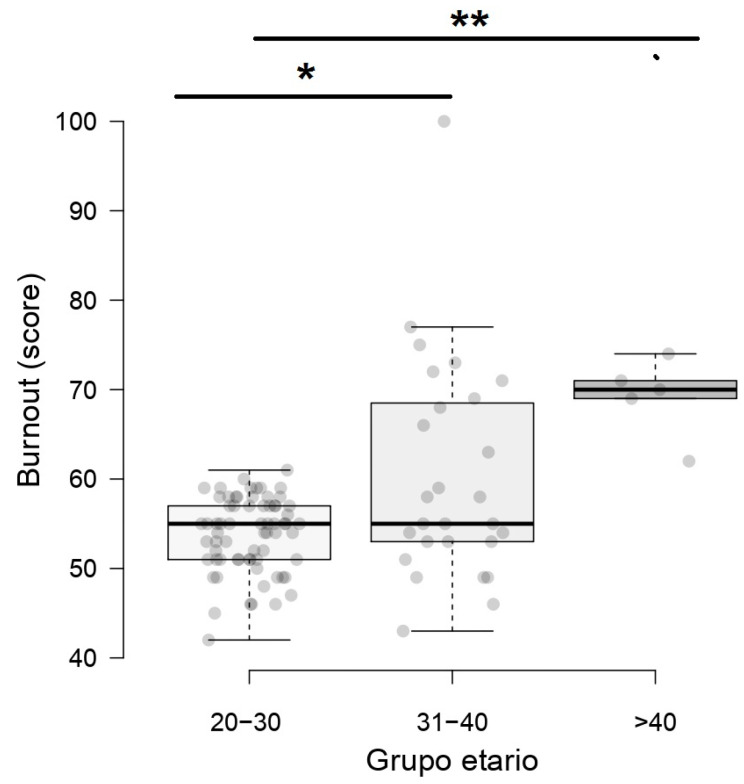
Distribution of BS according to the age group of nurses during the COVID-19 pandemic. * *p* < 0.05, ** *p* < 0.001.

Our results showed that the younger nurses had more BS symptoms (*p* < 0.001). In the age group from 20 to 30 years, the average BS was 53.8 ± 4.18 points (95% CI 52.79 to 54.8), while in the group from 31 to 40 years and 41 to 50 years, it was 60.3 (SD 12.35) points (95% CI 55.64 to 65) and 69.2 ± 4.44 points (95% CI 65.31 to 73.1), respectively (Figure 2). We show differences between nurses from 10 to 20 working years and older nurses (*p* < 0.05).

The multidimensional analysis of the BS is shown in Table 2. We found that 100% of nurses from 20 to 30 years old reported low emotional exhaustion, followed by 85% of nurses from 31 to 40 years old. Amounts of 1% and 38% of nurses aged 20–30 showed respectively high and moderate depersonalization, while 60% of the same group and 55% of nurses from 31 to 40 years old showed low depersonalization.

A total of 75% and 77% of nurses from 20 to 30 years old and 31 to 40 years old presented high personal achievement, respectively. However, only 3% of those in the second group had low levels. Predictive analysis showed that only age was a significant predictor of BS development (*p* < 0.001) (Table 3).

## 4. Discussion

This study assessed BS symptoms among nurses in a tertiary hospital and found lower levels of job fatigue in terms of emotional exhaustion and depersonalization. Likewise, the frequency of BS was higher in nursing professionals with a working time from 1 to 10 years, which is consistent with previous studies in American and European cohorts [2,14].

The strengths of the study are, first of all, taking as a population nurses from medical units specialized in patients with COVID-19, since they have had a great deal of responsibility and work since the beginning of the pandemic, guaranteeing patient safety and their continued care 24 h a day [10]. On the other hand, in two systematic reviews carried out worldwide on BS in nurses during the pandemic, Latin American studies have not been included [6,7]; therefore, this study would come to be one of the few contributions that have been made in South America. In addition, we used a standardized instrument based on statements of feelings and attitudes among health professionals to measure emotional exhaustion at work [21].

Our results showed that 95% of nurses reported low levels of emotional exhaustion, 57% reported low levels of depersonalization, and 76% reported high levels of personal fulfillment. These results agree with what was found in two hospitals in Libya where, during the COVID-19 pandemic, 67.1% of HCWs reported emotional exhaustion, 47.4% depersonalization, and, in the third dimension, their data were lower than ours since only 22.7% felt personal fulfillment.

This was repeated to a certain extent in other studies, such as the one carried out in two cities in China, where emotional exhaustion affected 78.5% and depersonalization affected 92.5%; the most worrying finding is that 48.6% experienced a serious lack of personal fulfillment. These results agree with what was found in two hospitals in Libya where, during the COVID-19 pandemic, 67.1% of HCWs reported emotional exhaustion, 47.4% depersonalization, and, in the third dimension, their data were lower than ours since only 22.7% felt personal fulfillment [24]. This was repeated to a certain extent in other studies, such as the one carried out in two cities in China, where emotional exhaustion affected 78.5% and depersonalization affected 92.5%; the most worrying finding is that 48.6% experienced a serious lack of personal fulfillment [25].

In Latin America, moderate and severe levels of BS are seen in medical and nursing personnel. In a pediatric hospital in Brazil, it was reported that nurses who worked with adolescents showed a higher frequency of BS compared to those who did not work with this group (77% vs. 32%, respectively) [26]. However, this was shown to a lesser extent in a multicenter study in which ICU nurses had 28.8% emotional exhaustion, 39.9% depersonalization, and 26.1% low professional achievement [27]. On the other hand, an Ecuadorian study reported that 63% of caregivers reported moderate emotional exhaustion, the same 65.7% reported depersonalization, and 78.1% reported personal fulfillment [28]. A possible explanation of these results could be due to the different strategies adopted in the care of patients with COVID-19 in each country and the epidemiological impact of SARS-CoV-2 outbreaks since the spread of variants and the accelerated increases in infections have played a key role in the dynamics of care in hospitals in the Americas region [29].

On the other hand, when we analyzed some studies in Peru, we found differences, for example, another tertiary hospital in Lima found that 68.3% and 17.5% of nurses reported feeling low and moderate emotional exhaustion, respectively. These results are lower if we compare them with 95% and 4% of our participants who felt low and moderate emotional exhaustion, respectively. In addition, we had similar results in the depersonalization dimension (57% vs. 60.3% of the low level and 34% vs. 33.3% of the medium level); however, we disagreed in the personal fulfillment dimension, where we evidenced differences in the high levels (41.3% vs. 76%) and moderate levels (33.3% vs. 23%) [30].

On the other hand, this reality is different from that of health centers in other regions, such as the Andes and the jungle. The study by Burgos and Salazar [31] showed that nurses in Northern Peru had high levels of stress (62% and 65% at high and medium levels, respectively) due to the high demand for personnel and scarce material resources. In this cohort, nurses exhibiting emotional exhaustion were also observed to be 55% highly and 32% moderately stressed, while those with depersonalization were 73% stressed. At this point, we emphasize the importance of conducting interregional studies because, despite strategies for the prevention and control of COVID-19, the health realities facing the pandemic vary, as demonstrated by previous studies [32,33,34].

Unfortunately, Latin America and the Caribbean have accounted for 32.1% of the total deaths from COVID-19 worldwide (1,440,853 deaths as of 31 August 2021) [35]. In Peru, during the second wave of COVID-19 in 2021, there will be an average of 680 deaths per day, with 214,480 deaths registered by August 2022 [12]. This number of deaths worldwide and an overload of work among nursing professionals, who in various countries suffer from a shortage of personnel and dissatisfaction in their jobs, can influence the abandonment of their profession, affecting the service provided to the population. A recent study in Brazil found that 29.1% of nurses, 22.9% of nursing technicians, and 15.7% of nursing assistants intended to leave their professions due to a lack of institutional support, a high workload, and skin injuries from the use of personal protective equipment [16]. This scenario must be quantified in each hospital, and each reality is always necessary to establish a link between BS and epidemiological outbreaks and adverse events among nursing professionals in order to establish better work support guidelines.

### 4.1. Limitations

First, because the design was unicentric, it was not possible to describe the differences between other local and regional realities, as resources may not be equal across provinces and health centers in rural or indigenous areas. This is especially important because, as some studies have described, these factors increase the likelihood of having BS in nursing [26]. Another limitation is that this study was focused on finding out the occupational factors of BS, but it is important to characterize its relationship with factors of mental health problems such as anxiety or depression, which have flourished during the pandemic [13,32,36].

### 4.2. Practical Implications

The study found that younger nurses (aged 20–30 years) are more susceptible to burnout. Therefore, interventions such as counseling, mentoring, and training programs could be developed to support younger nurses and help them cope with the demands of their job. Our finding highlights the importance of improving the work environment to prevent burnout. This could include changes to the work system and organization, improving interpersonal relationships, and promoting a culture of self-care. 

Additionally, providing adequate personal protective equipment (PPE) and other resources to prevent infection could help alleviate stress and anxiety in nurses working with COVID-19 patients [14,24,25,32,37]. This could also include training and education on infection prevention and control.

Healthcare organizations must regularly monitor for burnout among nurses and other healthcare workers. This could be carried out through regular surveys or check-ins to identify early warning signs of burnout and provide support as needed. On the other hand, this study found that males were more affected by burnout than females. Therefore, strategies should be developed to address gender disparities in the workplace and ensure that all nurses are supported equally.

## 5. Conclusions

We documented low levels of BS among nursing staff in healthcare units during COVID-19. Nonetheless, it was observed that work burnout occurred more frequently in those with shorter working periods (ranging from 1 to 10 years), and they were the only ones who showed high levels of emotional exhaustion and depersonalization, as well as low personal fulfillment.

The SB has many counterproductive effects on nursing staff, especially in the situation brought about by the pandemic, for which our health system was not prepared. Given this, it is imperative to develop strategies that apply not only to personnel but also to health entities, since their general status (infrastructure and resources) affects the emergence of illness. We must pay attention to the mental health of nurses through psychological assessment in order to carry out early intervention for their diseases. In addition to promoting teamwork and constant communication to reduce stress and improve employee efficiency. Likewise, institutions must have the necessary material and human resources to respond to the COVID-19 crisis [38,39].

## Figures and Tables

**Table 1:** Demographic, occupational, and clinical characteristics of nurses with Burnout Syndrome during the COVID-19 pandemic. N = 100.

Variable	Categories	Burnout		N (%)
		Moderate	High	
Age group (years)	20 to 30	68 (100)	0 (0)	68 (68)
	31 to 40	19 (70.4)	8 (29.6)	27 (27%)
	>41	1 (20)	4 (80)	5 (5%)
*p-value*		<0.001		
Sex	Female	67 (85.9)	11 (14.1)	78 (78%)
	Male	21 (95.4)	1 (4.6)	22 (22%)
*p-value*		0.882		
Working time (years)	1 to 10	53 (91.4)	4 (1.6)	57 (57%)
	11 to 20	27 (77.1)	8 (32.9)	35 (35%)
	21 to 30	8 (100)	0 (0)	8 (8%)
*p-value*		0.239		
SARS-CoV-2 Infection	Yes	35 (85.4)	6 (14.6)	41 (41%)
	No	53 (89.8)	6 (10.2)	59 (59%)
*p-value*		0.716		

**Table 2:** Multidimensional analysis of the Burnout Syndrome in nurses during COVID-19. Data in N (%).

Dimension	Frequency	Age Group (Years)		
		20 to 30	31 to 40	41 to 50
Emotional exhaustion	Low	68 (100)	23 (85.1)	4 (80)
	Moderate	0 (0)	3 (11.1)	1 (20)
	High	0 (0)	1 (3.7)	0 (0)
Depersonalization	Low	41 (60.2)	15 (55.5)	0 (0)
	Moderate	26 (38.2)	6 (22.2)	2 (40)
	High	1 (1.4)	6 (22.2)	3 (30)
Personal achievements	Low	0 (0)	1 (3.7)	0 (0)
	Moderate	17 (25)	5 (18.5)	1 (20)
	High	51 (75)	21 (77.7)	4 (80)

**Table 3:** Binary regression for burnout syndrome in nurses from COVID-19 medicine units. * *p* < 0.05 (significant).

Variables	Burnout			
	B	SE	*p*-Value	95% CI
Age (years)	7.57	1.32	<0.001 *	4.95 to 10.18
Sex	3.28	1.88	0.085	−0.46 to 7.01
Working time	0.65	1.19	0.585	−1.71 to 3.01
Previous SARS-CoV-2 infection	−0.57	1.44	0.691	−3.42 to 2.28

## Data Availability

The data presented in this study are available on request from the corresponding author.
